# TAGET: a toolkit for analyzing full-length transcripts from single molecular sequencing

**DOI:** 10.1093/bib/bbaf631.028

**Published:** 2025-12-12

**Authors:** Yuchao Xia, Susheng Miao, Ruibin Xi

**Affiliations:** College of Science, Beijing Information Science and Technology University, Beijing, 100192, China; Department of Head and Neck Surgery, Harbin Medical University Cancer Hospital, Harbin, 150081, China; School of Mathematical Sciences, Peking University, Beijing, 100871, China; Center for Statistical Science, Peking University, Beijing, 100871, China

## Abstract

Single-molecule Real-time Isoform Sequencing (Iso-seq) of transcriptomes by PacBio can generate very long and accurate reads, thus providing an ideal platform for full-length transcriptome analysis.A number of computational tools have been developed for long-read sequencing data. However, integrated computational frameworks for analyzing Iso-seq data are still lacking. We present a Toolkit for Analyzing full-length GEne Transcripts (TAGET) for Iso-seq. Starting from polished high-quality transcripts (circular consensus sequences or CCSs), TAGET first aligns transcripts to the reference genome by integrating alignment results from long and short reads and further improves splice site predictions using a Convolutional Neural Network (CNN). TAGET then annotates transcripts by comparing with reference isoform databases and classifies transcripts into seven classes. Finally, TAGET estimates gene or isoform expressions and performs differential expression gene (DEG) and differential isoform usage (DIU) analysis. We evaluate the performance of TAGET using a public Iso-seq dataset and newly sequenced Iso-seq datasets from tumor patients. TAGET gives significantly more precise novel splice site prediction and enables more accurate novel isoform and gene fusion discoveries, as validated by experimental validations and comparisons with RNA-seq data. We identify and experimentally validate a differential isoform usage gene ECM1, and further show that its isoform ECM1b may be a tumor-suppressor in laryngocarcinoma. Our results demonstrate that TAGET provides a valuable computational toolkit and can be applied to many full-length transcriptome studies.

